# Mimotopes for Mycotoxins Diagnosis Based on Random Peptides or Recombinant Antibodies from Phage Library

**DOI:** 10.3390/molecules26247652

**Published:** 2021-12-17

**Authors:** Wei Sun, Yan Zhang, Zhigang Ju

**Affiliations:** 1 Key Laboratory of Plant Physiology and Development Regulation, School of Life Science, Guizhou Normal University, Guiyang 550001, China; sunwei889@163.com (W.S.); 15186346268@163.com (Y.Z.); 2 Pharmacy School, Guizhou University of Traditional Chinese Medicine, Guiyang 550025, China

**Keywords:** mycotoxins, phage display, scFv, anti-idiotypic nanobody, simultaneous determination

## Abstract

Mycotoxins, the small size secondary metabolites of fungi, have posed a threat to the safety of medicine, food and public health. Therefore, it is essential to create sensitive and effective determination of mycotoxins. Based on the special affinity between antibody and antigen, immunoassay has been proved to be a powerful technology for the detection of small analytes. However, the tedious preparation and instability of conventional antibodies restrict its application on easy and fast mycotoxins detection. By virtue of simplicity, ease of use, and lower cost, phage display library provides novel choices for antibodies or hapten conjugates, and lead random peptide or recombinant antibody to becoming the promising and environmental friendly immune-reagents in the next generation of immunoassays. This review briefly describes the latest developments on mycotoxins detection using M13 phage display, mainly focusing on the recent applications of phage display technology employed in mycotoxins detection, including the introduction of phage and phage display, the types of phage displayed peptide/recombinant antibody library, random peptides/recombinant antibodies-based immunoassays, as well as simultaneous determination of multiple mycotoxins.

## 1. Introduction

Mycotoxins are nonvolatile and relatively low-molecular weight secondary metabolites produced by a variety of microscopic fungi. As mycotoxins are natural contaminants that exist in cereal, vegetables, milk and herbal medicine, they cannot be completely eliminated without damaging food. For fungi, mycotoxins are beneficial and play important roles in eliminating other microorganisms or invading host tissues. However, for animals, mycotoxins are acutely or chronically toxic, which interfere with absorption and metabolism of nutrients, resulting in the damage of endocrine and neuroendocrine functions, or suppression of the immune system [1,2,3]. Hence, stringent regulations relating to mycotoxins have been established to protect the consumer from their harmful effects [4].

To date, many food regulatory authorities have set maximum residue limits (MRLs) for mycotoxins, including EU, USA, China, etc. Even regulated mycotoxins and commodities, as well as MRLs, vary significantly in different countries; the request for analytical methods for mycotoxins detection is a worldwide priority. In order to meet the requirements of these regulations, many analytic methods for identification and quantification of mycotoxins have been developed, such as high performance liquid chromatography (HPLC) [5], liquid chromatography coupled with mass spectrometry (LC-MS) [6], gas chromatography coupled with mass spectrometry (GC-MS) [7,8], and so on. Although these methods provide excellent accuracy and reproducibility, there are still special limitations in actual practice, such as being relatively high cost, time-consuming, requiring of a skilled technical personnel, and any of these weaknesses make them unsuitable for quick and easy detection.

Alternatively, by the specific interaction of antibodies to mycotoxins hapten, immunoassays have particularly attractive properties in mycotoxins detection, for example, low cost, strong specificity, high sensitivity, fast test and simple operation [9]. Moreover, antibodies as one of the biorecognition elements, can exploit antibody-antigen interaction for specific detection of a particular analyte from complex matrices. Depending on the principles of detection, immunoassay can be mainly divided into four formats, including direct immunoassay, indirect immunoassay, sandwich immunoassay, and competitive immunoassay. Among these assays, sandwich immunoassay is mostly applied for detection of macromolecules, and competitive immunoassay is usually used for the detection of small molecules. The competitive immunoassay is frequently applied for low molecular weight mycotoxin detection, basing on the competitive binding between anti-mycotoxin antibody and mycotoxin conjugates. Until now, many portable immunoassay techniques have been developed for detection of small analytes. For instance, the lateral flow immunochromatographic assays (LFIAs), optimal suited for on-site test formats, require only the addition of the sample and can result in a readable signal [10]. The critical biochemical reagents in competitive immunoassay are the used antibody and hapten-conjugate. Nevertheless, the prepare processes of antibodies and the traditionally used hapten-protein conjugates are complicated, time-consuming, and expensive, which partly restricts their wide range of application.

On the one side, a large number of antibodies against various mycotoxins have been produced, such as monoclonal antibodies, polyclonal antibodies, and recombinant antibodies [11,12,13]. Nowadays, antibodies continue to be the predominant immunoreagent and some improvements of preparation have been introduced [14,15]. While validation of antibodies is often lacking, which may be a major limitation considering the quality and consistency of antibody-based technologies [16]. Meanwhile, the traditional antibodies need animal immunizations, longer time and larger expenses, which also influence their widespread use. On the other side, haptens cannot elicit an immuno response, so many artificial antigens conjugated with proteins were produced, like bovine serum albumin (BSA). However, mycotoxins conjugated with proteins still keep toxicity, and may produce toxic effects on the operators. Furthermore, analyte conjugation can unfavorably affect antibody recognition, or the release of the analyte moiety from the conjugate might even cause false positive results [17,18]. In addition, antigen-conjugates are not suitable for a large scale production with low cost. For example, the expenses of artificial antigen of fumonisin (FB_1_)-BSA, zearalenone (ZEN)-BSA and ochratoxin (OTA)-BSA were $193.344, $20.858 and $77.427, respectively [19].

Peptide mimics and recombinant antibodies are interesting alternatives to overcome above limitations. Therefore, more and more researchers have shifted the emphasis to alternatives of antibodies and mycotoxin conjugates. In general, there are two ways. One conventional approach is to generate anti-idiotype antibodies (AIds), which are raised against the variable regions of the original antibody or mycotoxins. For example, some AIds against various mycotoxins have been prepared successfully by monoclonal, polyclonal, or alpaca nanoantibody technology, such as FB_1_, aflatoxin (AFB_1_), deoxynivalenol (DON) [20,21,22,23].

Another approach to develop AId is via the phage displayed peptide technology. Phage-displayed peptide, which can mimic the antibody binding site on the antigen, has been demonstrated to be an alternative to the specific recognition for various targets. Phage-displayed peptides have been used in a number of applications, containing epitope mapping [24,25], molecular imaging [26], targeting drug delivery [27,28] and defining the protein-protein interactions [29,30]. Mimotopes for mycotoxins have also been selected through using phage display technology [31,32,33,34]. The mimotopes have been utilized for the detection of mycotoxins in the form of phage themselves or synthetic peptides. Moreover, combined toxic effects of mycotoxins have co-existed in agro-products [35]. So multiplexed detection of target analytes are of great significance owning to the advantages of time-saving, cost-effective, and high-throughput screening. This review focuses on the recent applications of phage display technology using for toxin detection, including the introduction of phage and phage display, the types of phage displayed peptide/recombinant antibody library, random peptides/recombinant antibodies-based immunoassays and simultaneous determination of multiple mycotoxins.

## 2. M13 Phage Display Technology

### 2.1. The Structure and Life-Cycle of M13 Bacteriophage

**Figure 1:**
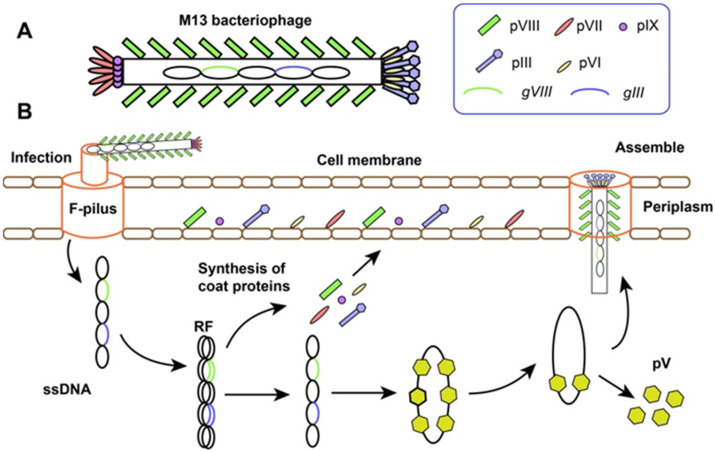
The basic structure (**A**) and life cycle (**B**) of filamentous bacteriophage.

M13 phage is one kind of rod-like filamentous phages with 1 μm long and 6 μm in diameters. As the demonstration in Figure 1A, a single-stranded DNA (ssDNA) was encapsuled in a protein tube, which composed mainly of systematically arranged molecules of pVIII (~2700 copies), named major coat protein. Meanwhile, 5 copies of pIII and pVI are located at one tip of the particle,5 copies of pVII and pIX are presented at the other tip, which respectively formed the “head” and “tail” of phage, named minor coat protein [36].

Filamentous bacteriophage can produce vast progenies in a short time without killing the host, which is different from lytic and lysogenic phages (Figure 1B). Relying on the adhesion between F pilus of *E. coli* bacteria and N-terminal domain of pIII, the whole phage is drawn into the periplasm of the cell. Only ssDNA is injected into the cytoplasm, and leaving the coat proteins outside. Taking advantage of the host machinery, a double-stranded replicate form (RF) DNA is synthesized, which provides the template for the transcription of phage genes and duplication of progeny ssDNAs. Then, all 5 coat proteins containing signal peptides are synthesized and secreted into the periplasm, where intact progeny phages are assembled successfully. The ssDNA carrying pV dimers produces packaging signal, which initiates the self-assembly. The “head” is formed first, while the “tail” is synthesized at last. The length of phage and number of pVIII are all determined by the size of ssDNA [37,38,39]. During the process of self-assembly, coat proteins arranged ordered in a special pattern. For example, pVIII always retains its N-terminal part outward, which allows foreign peptides or proteins display on the surface of phage particles.

### 2.2. Phage Display Technology

Phage display is a technology that can display foreign proteins or peptides on the surface of phage rods, either on the backbone or on both ends, which can achieve by targeted insertion of DNA sequence encoding foreign proteins or peptides. More importantly, foreign proteins or peptides still retain their ability to recognize the molecular targeting binding site. As early as in 1985, the restriction endonuclease *Eco*R I was first fused to pIII as recombinant minor coat proteins by Smith [40]. Subsequently, pVIII and pVI coat proteins can also be utilized for phage display [41,42,43] and even double display system is innovatively proposed [44].

Vectors of M13 bacteriophage used in phage display can be classified into different types, including type 3 + 3, type 6 + 6 and type 8 + 8 vectors. Among them, the foreign peptides encoded by the phagemid genome are named phagemid vectors. While the foreign peptides are encoded by phage genome are called phage vectors. Based on the number of displayed foreign peptides, phage vectors mainly classified into type 3/8 vectors and type 33/88 vectors, in which the displayed peptides fused to all copies of coat proteins or a fraction of them. Absolutely, some novel phage display systems have also been developed based on above phage or phagemid vectors. As reported by Wang et al. [44], magnetic nanoparticle-binding peptides and anti-sap2 antibody-binding peptides were separately displayed on pVIII and pIII to form a bi-functional nano-fibers using for the detection of *Candida albicans*. Meanwhile, various phage libraries comprising about 10^9^ variants are constructed, such as random peptide phage library [45], antibody phage library [46] and cDNA phage library [47], which lay a foundation for the study of bio-panning of targeted binding peptides and enzyme evolution. Next, we will review the main types of phage display libraries reported with respect to mycotoxins detection and discuss their construction methods, applied ranges, strengths and weaknesses.

## 3. Random Peptide Using for Mycotoxins Detection

### 3.1. Random Peptide Libraries

Random peptide libraries are the most common type of phage display library. Originally, random peptide, also called mimotopes, was applied to discover antibody-binding ligands whose specificity is not known in advance [48]. By direct insertion of peptide cDNA between the signal peptide and the N-terminus of the coat protein pIII, degenerate oligonucleotides can be introduced into the phage genome [39]. Then, random peptides were displayed on the surface of phage along with the propagating of phage particles. Furthermore, the detailed construction methods of random peptide libraries had been deeply reviewed by Kehoe and Kay [49]. According to the structure and length of peptide, a linear random peptide library varying in length from 6 to 43 amino acids and a loop random peptide library were constructed, respectively. Notwithstanding many random peptide libraries had been created for now, only few libraries were developed into a commodity for sell, such as Ph.D.™-12 Phage Display Peptide Library and Ph.D.™-7 Phage Display Peptide Library Kit.

A random peptide library may display tens of millions of peptide epitope, which makes phage display derived products play a significant role in the diagnosis and treatment of diseases. Macromolecules, bacteria, cells, tissues, organs, animals, and even nanoparticles [50,51,52,53,54,55] can all serve as the targets for bio-panning to obtain specific binding peptides. Therefore, many peptide mimotopes of mycotoxins have been identified through affinity selection from phage display libraries (Table 1), such as OTA, AFB_1_, FB_1_ and DON et al. In order to obtain mimotopes with higher affinity, a second generation peptide library had been constructed based on the identical sequence from the initial random peptide library. He et al. screened OTA from second-generation peptide library, which could improve the sensitivity approximately 10-fold [56].

Moreover, several methodologies have been successfully developed for small molecule-peptide/protein interaction studies based on the inter-disciplinary of biology, chemistry, physics, etc. For example, through detecting changes of the surface plasma signal excited by polarized light before and after binding to the immobilized molecule, surface plasma resonance (SPR) [65,66] can be most popularly applied for the affinity conformation between peptides and small molecules. In addition, circular dichroism (CD), isothermal calorimetry (ITC), and docking simulation are also utilized [67,68,69,70].

### 3.2. Random Peptide-Based Mycotoxins Detection

Compared with other analytic methods, immunoassays have several advantages for rapid test, including higher sensitivity, stronger specificity, facile sample preparation, and ease of use. As one of immunoassays, ELISA has been used widely for mycotoxin detection following the development of monoclonal and polyclonal antibodies. However, the low efficiency of chemical conjugation of mycotoxins to a carrier protein may result in substantial bridge group interference and cross-reactions [31]. So protein or peptide mimics as immunochemical reagents have been developed as one possible alternative of mycotoxins.

**Figure 2:**
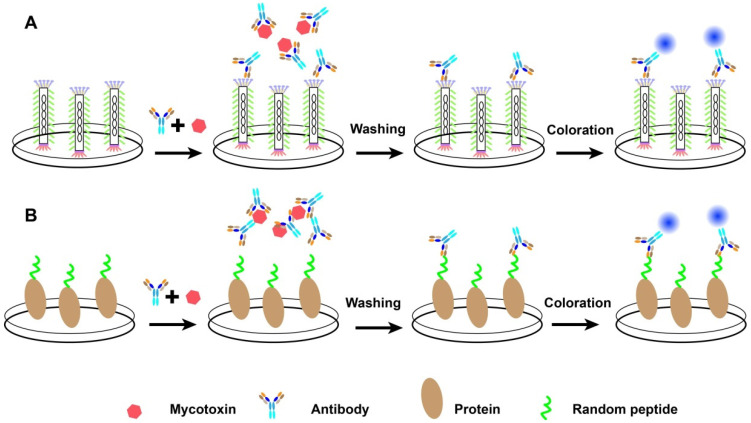
The process of competitive immunoassays based on phage form (**A**) or peptide fusion protein (**B**).

Due to the tiny size, random peptides against a special mycotoxin or antibody cannot be directly immobilized on the solid surface for immunoassay, except in one report where the synthetic peptide alone was sufficient for binding to the antibody [31]. Actually, random peptides were always conjugated with some proteins to form fusion proteins using for immunoassays. The most direct way, M13 bacteriophage displayed with target peptides were used as coating antigens. For example, one mimotope peptide P3 (HPSDPRH), the AFB_1_ mimotope peptide, was obtained from Ph.D.™-7 Phage Display Peptide Library [58]. The recombinant phage was applied for the detection of AFB_1_ through an indirect competitive ELISA. Compared with a conventional indirect competitive ELISA with the AFB_1_-BSA conjugate, there was no special difference between ELISA methods in accuracy and precision. Similarly, for another example, 5 mimotopes against MAb 24 specific to aflatoxins B_1_ were identified from a random 8-peptide library [33]. The whole phage displayed with mimotope peptides were also used in an indirect competitive ELISA for analyzing total aflatoxin concentration with an IC_50_ value of 14 μg/kg and the linear range of 4–24 μg/kg (Figure 2A). Moreover, phage-based dot-immunoassay device based on PVDF membrane strips were also developed and acted as alternatives of 96-well plate [60].

However, phage-based immunoassays were not suitable for diagnosis due to the following reasons: (1) Phages with filamentous nature are “unconventional” reagents which can infect *E. coli*. (2) Peptide linked to the phage particles leads to complex difficulties in measuring the phage displayed peptide and quality control. (3) Peptide concentration was not controlled precisely, with the avidity artifacts associated with pentavalent display on the phage [22]. So mimotope conjugated with special proteins named fusion proteins were prepared and used as coating antigens in the immunoassay for analyzing mycotoxins (Figure 2B). For example, a phage clone that recognized anti-fumonisin McAb 1D11 from a phage random loop constrained heptapeptide library was selected as mimotope peptide and conjugated with bovine serum albumin as coating antigen [61]. The results for fumonisin detection showed that the linear range of the inhibition curve was 1.77–20.73 ng/mL and the limit of detection was 1.18 ng/mL. In another example, phage displayed peptide which bind to ani-FB_1_ antibody from a 12-mer peptide library was selected and conjugated with maltose binding protein (MBP) to form fusion protein [62]. The fusion protein was used as a coating antigen to develop a qualitative Elispot assay with a cutoff level of 2.5 ng/mL, and the results was 10-fold more sensitive than that of measurement from chemically synthesized FB_1_-BSA conjugates based Elispot immunoassay. In addition, an on-chip binding inhibition assay based on A2 peptide (VTPNDDTFDPFR) conjugated with biotin was developed for the detection of FB_1_ through microassay [63].

Beyond that, colloidal gold strip as a rapid and inexpensive detection method was also used for rapid detection of mycotoxins with the help of mimotope peptide. Lai et al. [71] developed a colloidal gold strip using chemically synthesized gold nanoparticles conjugated with anti-OTA monoclonal antibodies and the OTA mimotope was screened from a Ph.D.™-7 phage display peptide library. The results revealed that 10 ppb of OTA was detected in 10 min, which provides a rapid method without using the mycotoxin.

## 4. Recombinant Antibody Using for Mycotoxins Detection

### 4.1. Recombinant Antibody Libraries

**Figure 3:**
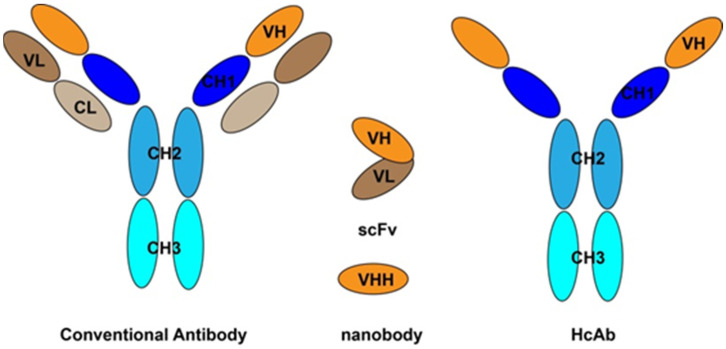
Different types of recombinant antibodies compared with conventional antibody.

Recently, the recombinant antibodies devoid of light chain have emerged as a salient alternative for immunosensing. Compared with conventional antibodies, the recombinant antibodies are smaller, such as antigen binding fragment (Fab), heavy chain only antibody (HcAb), single chain fragment variable (scFv) and a single domain heavy chain antibody (VHH, termed “nanobody”, Figure 3). Specially, the recombinant antibodies are particularly apt for genetic manipulation such as large-scale amplification and antibody-protein fusion to create bi-functional molecules [72]. Depending on the preparation method, there are mainly two types of recombinant antibody libraries, naive libraries and immune libraries. 

Naive libraries are created from rearranged variable region (V) gene pools of a non-immunized individual [39]. Without immunity, the antibody structure, codon usage, and sequence diversity of naive libraries can be designed as desired. Most importantly, the library is not biased to small antigens. Additionally, good-quality antibodies against conserved antigens were also yielded from naive libraries [73]. In addition, high-affinity antibodies against mycotoxins have been isolated from naive libraries, such as AFB_1_, citrinin (CIT), ZEN, and DON [23,74,75,76,77]. However, for some special application, antibodies from naive libraries showed lower affinity, so immune libraries are generated by amplification variable region (V) genes of antibody extracted from plasma cells from immunized donors. Primarily, animals were immunized with target antigens for the isolation of recombinant antibodies. Subsequently, RNA extracted from spleen cells was transcribed into complementary DNA. Finally, a recombinant library was constructed through amplifying antibodies genes into appropriate vector. Overall, the above types of libraries are typically created to obtain an antibody for a specific target (Table 2) [78].

### 4.2. ScFv Antbibodies Based-Detection Method

The scFv first developed in 1988 is a recombinant protein composed of a variable region of heavy chain (VH) and a variable region of light chain (VL) of the antibody through a short peptide [79,80]. Due to the small molecular weight, strong penetration, and high affinity, scFv has been widely used in tumor therapy, infectious disease prevention and treatment, food safety residue detection, as well as other fields [81]. Without phage display, scFv can be generated from spleen cells directly and many of them have been prepared for mycotoxins detection, such as FB_1_, ZEN, DON, and CIT [82,83,84,85]. The scFv generated from phage recombinant antibody library were focus on the AB_1_ detection. For example, through biopanning to immobilized AFB_1_-BSA conjugate, two scFv fragments named YM1 C3 and TomI-F6 were generated from non-immunized Yamo 1 library and semisynthetic libraries (Tomlinson I & J). After analysis of binding sensitivity by competitive ELISA, the IC_50_ of YM1 C3 and TomI-F6 was 0.04 µg/mL and 0.14 µg/mL, respectively. Then, these two scFv DNA fragments were cloned into an AP expression vector to form scFv-AP fusions, and the competitive ELISA results showed that the binding sensitivity of YM1 C3-AP (IC_50_ = 0.034 µg/mL) is approximately 4 fold higher than that of TomI-F6-AP (IC_50_ = 0.14 µg/mL). So the YM1 C3-AP was used as a convenient one-step detection probe for competitive ELISA of AFB_1_ [86]. For diagnosis, the affinity and sensitivity of selected scFv to mycotoxins is very important. Hence, a lot of efforts were made to increase its affinity and sensitivity. Through panning antibodies against AFB_1_-BSA and AFB_1_, Moghaddam et al. found many of the antibodies isolated specifically bound AFB_1_-BSA, not soluble AFB_1_ or BSA [87]. At the same time, similar results were obtained by Chen et al. Compared to the selection against AFB_1_-bovine serum albumin conjugate, the isolated scFvs against AFB_1_ showed higher specificities for AFB_1_ [74]. Additionally, two high-quality scFv antibodiesagainst AFB_1_ were isolated from synthesized immune scFv library using 20 hybridoma cell lines by Li et al. Thedis IC_50_ of 1A7 and 2G7 was 0.02 ng/mL and 0.01 ng/mL, respectively [88]. Recently, antibody-ligand interactions were analyzed and improved by Rangnoi et al. through chain-shuffling technique using a naive human phage-displayed scFv library and a constructed VH/VL chain-shuffled library. One clone named sAFH-3e3 showing 7.5-fold improvement in sensitivity was obtained [89].

### 4.3. Anti-Idiotypic Antibody Based-Detection Method

Anti-idiotypic antibody is a secondary antibody that targets the idiotype of the primary antibody, which sit on the variable regions of immunoglobulins and possess specific antigenic determinants. Numerous anti-idiotypic antibodies against both large and small molecules have been developed and applied in diagnostics and immunoassays [94,95]. Interestingly, a type of antibody from camelids completely devoid of light chains (only heavy chain antibody) was found in 1993 [96]. The variable domain (VH) of such heavy chain antibodies is formed by only one variable domain (VHH), termed nanobody [97,98]. In virtue of small size, good physical and chemical properties, large scale in production, and easy manipulating, anti-idiotypic nanobodies have also been developed and used for diagnostic and therapeutic purposes [99,100].

Absolutely, anti-idiotypic antibodies were also applied in environmental immunoassays, including mycotoxins diagnostic. The first research using nanobodies for mycotixins detection was reported by Wang et al. In their work, the authors constructed an antibody phage library from the mRNA of an alpaca immunized with an anti-aflatoxin monoclonal antibody (MAb) 1C11 and isolated VHH antibodies, which applied to immunoassay towards aflatoxin as a coating antigen. The immunoassay with an IC_50_ of 0.16 ng/mL showed a good correlation (R^2^ = 0.89) towards the conventional ELISA method [78]. Normally, anti-idiotypics nanobodies mimics, selected from recombinant antibodies library or naive antibodies library, were acted as surrogate antigens which can competitively bind with homologous antibodies. Then the unbound antibodies with mycotoxins were captured by the pre-coated nanobodies and detected such as CIT, OTA, 15-acetyl-deoxynivalenol (15-acDON), ZEN [76,80,91,92,93,94].

**Figure 4:**
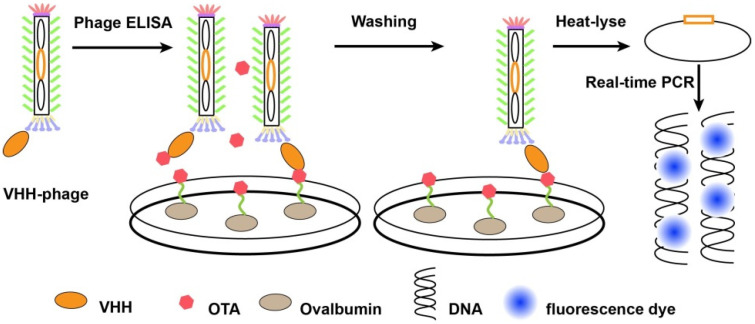
The process of PD-IPCR based on VHH-phage. Firstly, VHH-phages and OTA were mixed and incubated with ovalbumin conjugated OTA pre-coated on the solid surface. Then, VHH-phages binding with OTA were washed out and redundant VHH-phages were fixed. Finally, the DNA of VHH-phages were released by heat-lyse and used as templates of real-time PCR.

Besides competing immunoassay, other methods based on nanobodies were also developed. For instance, phage display-mediated immuno-polymerase chain reaction (PD-IPCR), a highly promising technique for ultrasensitive analysis of small molecules, was first described by Zhang et al. and has been applied for mycotoxins detection [101]. As signal output, PD-IPCR has been reported for ultrasensitive analysis of antigens combining nanobodies with phage DNA, including noncompetitive phage anti-immuno complex real-time (RT) PCR [102], phage-based open-sandwich immune-PCR [103] and competitive phage real-time PCR. For example, Liu et al. [91] constructed an alpaca-derived heavy-chain antibodies (VHH) library and obtained the clone-28 which showed the lowest 50% inhibitory concentration. Then, the VHH phage-based RT immuno-PCR was developed and utilized for the analysis of OTA (detailed process illustrated in Figure 4). The results displayed that detection limit of the VHH phage-based RT immuno-PCR was 3.7 pg/L with a linear range of 0.01–1000 pg/mL, indicating the reliability of VHH phage-based RT-IPCR in the detection of OTA in cereal samples. In the same way, anti-idiotypic VHH PD-PCR was also supplied for ultrasensitive determination of mycotoxin zearalenone in cereals. Compared with phage ELISA, the LOD of Z1 (anti-idiotypic VHH phage clone) based PD-IPCR was 12-fold improved, together with a detection limit of 6.5 pg/mL and a linear range of 0.01–100 ng/mL [76]. However, PD-IPCR method needs special instrument (Real time fluorescence quantitative PCR instrument) and a longer assay time (more than 3 h), and it is not suitable for on-site fast detection of mycotoxins.

**Figure 5:**
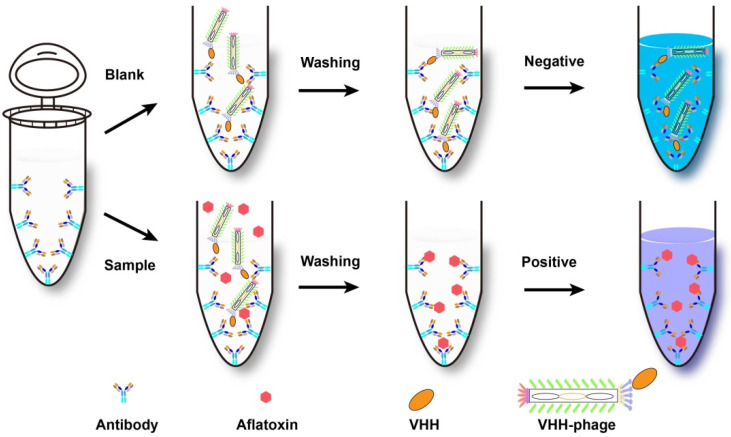
The detailed process of iLAMP assay (adapted from [107]).

As an alternative to PCR based analysis, the loop-mediated isothermal amplification (LAMP) is an innovative technique for rapid and easy detection of target nucleic acids. Since first reported in 2000, LAMP has been applied in various fields of diagnosis, such as pathogen detection and disease diagnosis [104,105,106]. Due to the higher specificity and efficiency, on-site testing, naked eye identification, and isothermal amplification, immune-LAMP (iLAMP) assay was exploited for aflatoxin detection. The basic process is as follows (Figure 5): Firstly, anti-aflatoxin mAb 1C11was pre-coated on the bottom of a PCR tube. Secondly, the sample extract and nanobody-phage V2-5 were added to the tube simultaneously with a competition binding between the phage and aflatoxin to mAb 1C11. Subsequently, the unbound phages were washed away after the incubation. Finally, the LAMP solutions were added into the tube for amplification for visual detection. The color of violet and sky blue respectively means positive and negative result. The visual detection limits of iLAMP of AFB_1_, AFB_2_, AFG_1_, and AFG_2_ in peanut samples were 1.6, 1.6, 3.2, and 16 µg/kg, respectively [107].

## 5. Simultaneous Determination of Multiplex Mycotoxins

Currently, many techniques have been developed to quantitatively or qualitatively detect multiple mycotoxins, including chromatographic techniques, immunochemical assays and electrochemical techniques [108,109,110,111,112]. For example, with the help of different mycotoxin or antibody conjugated fluorescent nanoparticles, multiplexed immunochromatographic assay (mICA) strips were widely developed for the simultaneous monitoring of multiple mycotoxins, such as gold nanoparticles [113,114], quantum dot microbeads [115], and amorphous carbon nanoparticles [116]. In the following sections, different multiplex immumoassay of mycotoxins using peptide mimotope or recombinant antibodies are presented.

### 5.1. Random Peptide-Based Multiplex Detection

Due to rapidity, good specificity, high throughput, convenience and low cost, ICA is the most commonly used and mature screening platforms for on-site determinations. ICA requires only the addition of the sample initiating a series of reactions which result in a readable signal. ICA had been developed for singleplex and multiplex detection based on traditional antibodies [117,118,119,120,121,122,123]. Recently, a multiplex ICA based on random peptides was developed for detection of three different mycotoxins. Yan et.al developed an economical and sensitive QDs and QBs based mICA for the rapid detection of FB_1_, ZEN, and OTA without the building-up process of mycotoxin conjugates [19]. QBs conjugated with anti-FB_1_ mAb or anti-ZEN mAb, and QDs coupled with anti-OTA mAb, were selected as fluorescent reporters. Furthermore, phage-displayed FB_1_, ZEN and OTA mimotope peptide were monovalently fused to MBP, which were applied onto the test line of the mICA as the mimetic coating antigen. The immunochromatograghic test is applied with the competition between mycotoxins in the sample and peptide mimics. The visual detection limits of peptide-MBP-based mICA could be obtained as 0.25 ng/mL for FB_1_, 3.0 ng/mL for ZEN, and 0.5 ng/mL for OTA within 10 min. The proposed mICA was comparable with UPLC-MS in terms of reliability in detecting FB_1_, ZEN and OTA.

### 5.2. Recombinant Antibodies-Based Multiplex Detection

#### 5.2.1. Time-Resolved Fluorescence Immunochromatographic Assay

Time-resolved fluorescence, which used lanthanides as tracers, has a longer fluorescence lifetime that could eliminate the background interference, thus achieving more sensitive and specific assays. Tang et al. prepared a novel Eu/Tb (III) nanosphere with enhanced fluorescence conjugated to anti-idiotypic nanobody and established a competitive time-resolved strip method for rapid, quantitative, and simultaneous detection of aflatoxin and zearalenone in maize and its products. The results showed that the half inhibition concentration was 0.46 and 0.86 ng/mL for AFB_1_ and ZEN, and the detection limit was 0.05 and 0.07 ng/mL, respectively [124].

#### 5.2.2. Duplex Real-Time PCR Methods

As demonstrated above, PD-IPCR has been an ultrasensitive immunoassay for mycotoxins detection. Through the combination of PD-IPCR and RT-PCR, a new detection platform was developed for simultaneously detecting of aflatoxins and Aspergillus section Flavi in stored maize. The quantitative standard curves for simultaneous detection of aflflatoxins and Aspergillus section Flavi were constructed, with detection limits of 0.02 ng/mL and 8 × 10^2^ spores/g, respectively. The entire process for the simultaneous detection requires less than 1 day. Therefore, this detection platform provides new ideas for simultaneous detection of small molecular contaminants and microorganisms [125].

## 6. Conclusions

Mycotoxins are small size secondary metabolites of fungi, which pose a threat to the safety of medicine, food and public health. Many novel methods have been developed for mycotoxins detection, such as antibodies and aptamers [126]. M13 bacteriophage has also been applied for screening mimotopes of small analytes including mycotoxins as target recognition element.

In the bio-panning of mimitopes, random peptides library and recombinant antibodies library were separately constructed with M13 phage display technology and applied to select desired surrogate antigens or antibodies. It should be noted that both libraries have their own advantages and disadvantages in the application, and a better choice should be made according to our own situation. Random peptide library can mimic continuous or discontinuous (distant in the primary sequence but close in the folded native conformation) determinants on ligands that specifically bind receptors or other proteins, and even nonproteinaceous ligands. Random peptide library shows no bias to small antigens, which makes it a good choice for mycotoxins detection. However, the peptides from random peptide library may show lower affinity, and recombinant antibody library will be a better choice because the recombinant antibodies generated from immune donors inherently have good affinities. Furthermore, after bio-panning, the recombinant antibody with the highest affinity can be successfully isolated.

In the application, individual peptides or phage form were rarely used. Instead, random peptides were always conjugated with other proteins to form fusion proteins and then applied for mycotoxins detection. Beyond that, with the help of multi-functional phage display technology, a versatile biosensor based on M13 phage has been assembled for detection analysis. For example, M13 phage was decorated with different mimotopes on the tip and backside of phage bodies, which endows it new features, such as the targeting bound capacity, the optical property of quantum dot, the accumulation of magnetic nanoparticle, and so on [127]. Compared with random peptides, recombinant antibodies can be directly immobilized on the solid surface to bind with antigens. So many recombinant antibodies, including scFvs and anti-idiotypic antibodies were isolated from recombinant antibody libraries and applied for sensitive diagnosis of various mycotoxins through ELISA, PD-IPCR and iLAMP, etc. Meanwhile, novel immunoregents using for the detection of different congeners from a mycotoxin group might be generated through recombinant antibodies in the future. This may be beneficial to detect broad range of mycotoxins. What is more delightful is that simultaneous detection of mycotoxins was developed based on the phage display. To some extent, all these results demonstrated the advantages of recombinant antibody in stronger specificity, higher sensitivity, less time consuming, and superior safety. However, there are still some challenges to select interested mimotopes binding to small molecule epitopes. This hampers the more extensive applications of phage display in mycotoxin detection. Therefore, efforts should be made to cope with the difficulties in the future.

## Figures and Tables

**Table 1:** Mycotoxins-binding mimotopes screened from phage display library.

Mycotoxin	Library	Passenger Protein	Peptide Mimics	Linear Range	LOD	Detection Principle	Sample Matrices	Ref.
DON	Random 7-mer peptide library	pIII	SWGPFPF; SWGPLPF	0.1–10 µg/mL	-	Competitive ELISA	Wheat	[31]
AFB_1_	Random 8-mer peptide library	pIII	-PHPWNP-; -T-HRNW-	4–24 µg/kg	-	Competitive ELISA	Peanut and feedstuff	[33]
	Random Cys-4/Cys-6 peptide library	pVIII	CYMD-C	-	-	Competitive ELISA	Groundnut	[57]
	Ph.D.™-7 Phage Display Peptide Library	pIII	HPSDPRH	100–2500 pg/mL	-	Competitive ELISA	Rice, wheat, corn, and feedstuff	[58]
OTA	Random 7-mer peptide library	pIII	GMVQTIF	0.005–0.2 ng/mL	0.1 ng/mL	Competitive ELISA	Corn	[32]
	Second generation peptide library	pIII	AETYGFQLHAMK	0.006–0.245 ng/mL	0.005 ng/mL	Chemiluminescent ELISA	Corn, rice, and instant coffee	[56]
	Ph.D.™-7 phage display peptide library	pIII	IRPMVXX	200–8000 pg/mL	150 pg/mL	Competitive ELISA	-	[59]
ZEN	Ph.D.™-7 Phage Display Peptide Library	pIII	DAVILLM; HHCHWWH	100–10,000 pg/mL	100 pg/mL	Competitive ELISA	Wheat, corn, and feedstuff	[34]
	Random 12-mer peptide library	pIII	ESYWATVPWTRH	50–100 µg/kg	-	Dot-immunoassay	Peanut, corn and rice	[60]
FB_1_	Ph.D.-C7C phage display peptide library	pIII	E-L-P-T-L	1.77–20.73 ng/mL	1.18 ng/mL	Chemiluminescent Immunoassay	Maize, feedstuff, and wheat	[61]
	Random 12-mer peptide library	pIII	NNAAMYSEMATD; TTLQMRSEMADD	-	0.21 ng/mL	Elispot Immunoassay	Maize, feedstuff, and rice	[62]
	Ph.D.™-12 Phage Display Peptide Library	pIII	VTPNDDTFDPFR	17.3–79.6 ng/mL	11.1 ng/mL	Microarray-based Immunoassay	Maize and wheat	[63]
Phomopsin	Random 15-mer phage display peptide library	pIII	CTVALCNMYFGAKLD	-	-	Competitive ELISA	Lupin seed	[64]

**Table 2:** Recombinant antibodies and their performances in immunoassay applications.

Mycotoxin	Antibody Type	Library	Linear Range	LOD	IC_50_	Detection Principle	Sample Matrices	Ref.
FB_1_	scFv	VHH library	2.10–76.45 µg/L	8.32 µg/kg	12.67 µg/L	Competitive ELISA	Corn	[82]
ZEN	scFv	VHH library	-	-	17 ng/mL	Competitive ELISA	Corn	[83]
DON	scFv	-	-	-	8.2 ± 0.6 ng/mL	Competitive ELISA	Wheat	[84]
CIT	scFv	Mutational phage library	25–562 µg/mL	14.7 ng/mL	120 ng/mL	Competitive ELISA	Corn	[85]
AFB_1_	scFv	Human non-immunized scFv library	0.007–0.2 µg/mL	0.007 µg/mL	0.034 µg/mL	Competitive ELISA	-	[86]
AFB_1_	scFv	Naive recombinant antibody libraries	-	-		Competitive ELISA	-	[87]
AFB_1_	scFv	Tomlinson libraries I + J			0.4 ng/mL	Competitive ELISA		[74]
AFB_1_	scFv	positive phage-display library	-	-	0.01 ng/mL	Competitive ELISA	-	[88]
AFB_1_	scFv	Variable VH/VL shuffled library	0.019–5 µg/mL		0.02 µg/mL	Competitive ELISA	-	[89]
CIT	VHH	Naive alpaca phage displayed VHH library	5–300 ng/mL	7.6 μg/kg; 8.6 μg/kg	44.6 ng/mL	VHH-based ELISA	Wheat, Rice	[75]
OTA	VHH	-	0.003–0.673 ng/mL	0.001 ng/mL	0.097 µg/mL	Competitive ELISA	Corn, rice, wheat	[90]
OTA	VHH	VHH Library	0.01–1000 pg/mL	3.7 pg/L,	0.31 ng/mL	PD-IPCR	Corn, wheat, rice	[91]
OTA	VHH	-	0.06–0.43 ng/mL	0.04 ng/mL	0.13 ng/mL	Fluorescencecompetitive ELISA	Rice, oats, barley	[92]
15-AcDON	VHH	VHH library	10–5000 ng/mL	19 ng/mL	0.5 µM	Competitive ELISA	-	[93]
ZEN	VHH	Naive alpaca phage displayed VHH library	0.11–0.55 ng/mL	0.08 ng/mL	0.25 ± 0.02 ng/mL	PD-IPCR	Corn, wheat, rice	[76]
